# Tracking NF-κB activity in tumor cells during ovarian cancer progression in a syngeneic mouse model

**DOI:** 10.1186/1757-2215-6-63

**Published:** 2013-09-10

**Authors:** Andrew J Wilson, Whitney Barham, Jeanette Saskowski, Oleg Tikhomirov, Lianyi Chen, Hye-Jeong Lee, Fiona Yull, Dineo Khabele

**Affiliations:** 1 Department of Obstetrics and Gynecology, Division of Gynecologic Oncology, Vanderbilt University Medical Center, B1100 Medical Center North, Nashville, TN 37232, USA; 2 Department of Cancer Biology, Vanderbilt University Medical Center, Nashville, TN, USA; 3 Vanderbilt-Ingram Cancer Center, Vanderbilt University Medical Center, Nashville, TN, USA

**Keywords:** NF-κB activity, Ovarian cancer, Syngeneic mouse model, Macrophages, Bioluminescence

## Abstract

**Background:**

Nuclear factor-kappa B (NF-kappaB) signaling is an important link between inflammation and peritoneal carcinomatosis in human ovarian cancer. Our objective was to track NF-kappaB signaling during ovarian cancer progression in a syngeneic mouse model using tumor cells stably expressing an NF-kappaB reporter.

**Methods:**

ID8 mouse ovarian cancer cells stably expressing an NF-kappaB-dependent GFP/luciferase (NGL) fusion reporter transgene (ID8-NGL) were generated, and injected intra-peritoneally into C57BL/6 mice. NGL reporter activity in tumors was non-invasively monitored by bioluminescence imaging and measured in luciferase assays in harvested tumors. Ascites fluid or peritoneal lavages were analyzed for inflammatory cell and macrophage content, and for mRNA expression of M1 and M2 macrophage markers by quantitative real-time RT-PCR. 2-tailed Mann-Whitney tests were used for measuring differences between groups in *in vivo* experiments.

**Results:**

In ID8-NGL cells, responsiveness of the reporter to NF-kappaB activators and inhibitors was confirmed *in vitro* and *in vivo*. ID8-NGL tumors in C57BL/6 mice bore histopathological resemblance to human high-grade serous ovarian cancer and exhibited similar peritoneal disease spread. Tumor NF-kappaB activity, measured by the NGL reporter and by western blot of nuclear p65 expression, was markedly elevated at late stages of ovarian cancer progression. In ascites fluid, macrophages were the predominant inflammatory cell population. There were elevated levels of the M2-like pro-tumor macrophage marker, mannose-receptor, during tumor progression, and reduced levels following NF-kappaB inhibition with thymoquinone.

**Conclusions:**

Our ID8-NGL reporter syngeneic model is suitable for investigating changes in tumor NF-kappaB activity during ovarian cancer progression, how NF-kappaB activity influences immune cells in the tumor microenvironment, and effects of NF-kappaB-targeted treatments in future studies.

## Background

Ovarian cancer is the most lethal gynecologic malignancy in the United States [1]. Most women diagnosed with epithelial ovarian cancers have advanced, metastatic disease characterized by abdominal ascites and peritoneal carcinomatosis [2]. Inflammation is a hallmark of cancer [3], and there is ample evidence linking chronic inflammation to pro-tumorigenic effects in ovarian cancer development and progression [4-9]. Pro-inflammatory cytokines and chemokines produced by peritoneal macrophages contribute to the distinct clinical features of ovarian cancer, specifically malignant ascites and widespread peritoneal tumor implants [4,10,11].

The nuclear factor-kappaB (NF-κB) pathway is a critical molecular link between inflammation and cancer. NF-κB signaling is known to play an important role in several malignancies, including ovarian cancer [9,12-16]. There are five known NF-κB subunits in humans: NF-κB 1 (p105/p50), NF-κB 2 (p100/p52), RelA (p65), RelB and c-Rel [17]. In a quiescent state, NF-κB subunits are bound to inhibitors of κB protein (IκBs) and are sequestered in the cytoplasm. Activation of the classical NF-κB cascade is initiated by growth factors, microbes, cytokines and genotoxic stress, which in turn activate the IκB kinase (IKK) complex. Activated IKK phosphorylates IκBα, which is then ubiquitinated. As a result, the p50-RelA dimer translocates to the nucleus, where it binds to the promoter/enhancer regions of genes involved in the regulation of cell growth, apoptosis and inflammation [16].

Several lines of evidence link NF-κB activity to ovarian cancer progression. Constitutive activation of NF-κB is observed in a large subset of ovarian tumors and is associated with tumor growth and hallmarks of progression [4,13-16]. Moreover, NF-κB is known to link a specific subset of pro-inflammatory cytokines and chemokines, including a TNF cytokine network, to human epithelial ovarian cancer [6,10,15,18]. Such functions of NF-κB have led to successful preclinical testing of NF-κB inhibitors in ovarian cancer model systems, for example thymoquinone, a product of the medicinal plant *Nigella sativa*[19]. However, most preclinical models are limited by the fact that drug effects are tested on cancer cells in the absence of the supporting tumor microenvironment, essential for cancer progression *in vivo*.

Ovarian tumors are known to polarize macrophages to display pro-tumorigenic characteristics in a NF-κB-dependent manner [4,20]. Classically activated or cytotoxic anti-tumorigenic macrophages (also called M1) and “alternatively” activated pro-tumorigenic macrophages (M2) represent two extremes in the spectrum of the macrophage phenotype [21]. This polarization is part of a complex interplay of signaling and responses between tumor cells and inflammatory cells such as macrophages, T cells and dendritic cells [22-24]. Despite current knowledge, substantial gaps remain regarding the specific influence of NF-κB activation in the peritoneum during ovarian tumor dissemination.

Herein, we describe the generation of mouse ID8 ovarian cancer cells stably expressing a green fluorescent protein (GFP)/luciferase fusion product under the control of a synthetic NF-κB-dependent promoter [25,26]. These reporter cells allow the intra-vital mapping of NF-κB activity during tumor progression in a syngeneic mouse model of ovarian cancer. Successful generation of this model provides unique insight into the role of NF-κB activation in different phases of ovarian cancer progression, effects of modulating NF-κB activity on host cell immune responses in the tumor microenvironment, and will serve as a powerful tool for pre-clinical testing of agents that target NF-κB in ovarian cancer.

## Materials and methods

### Generation of ID8-NGL cells

**Figure 1:**
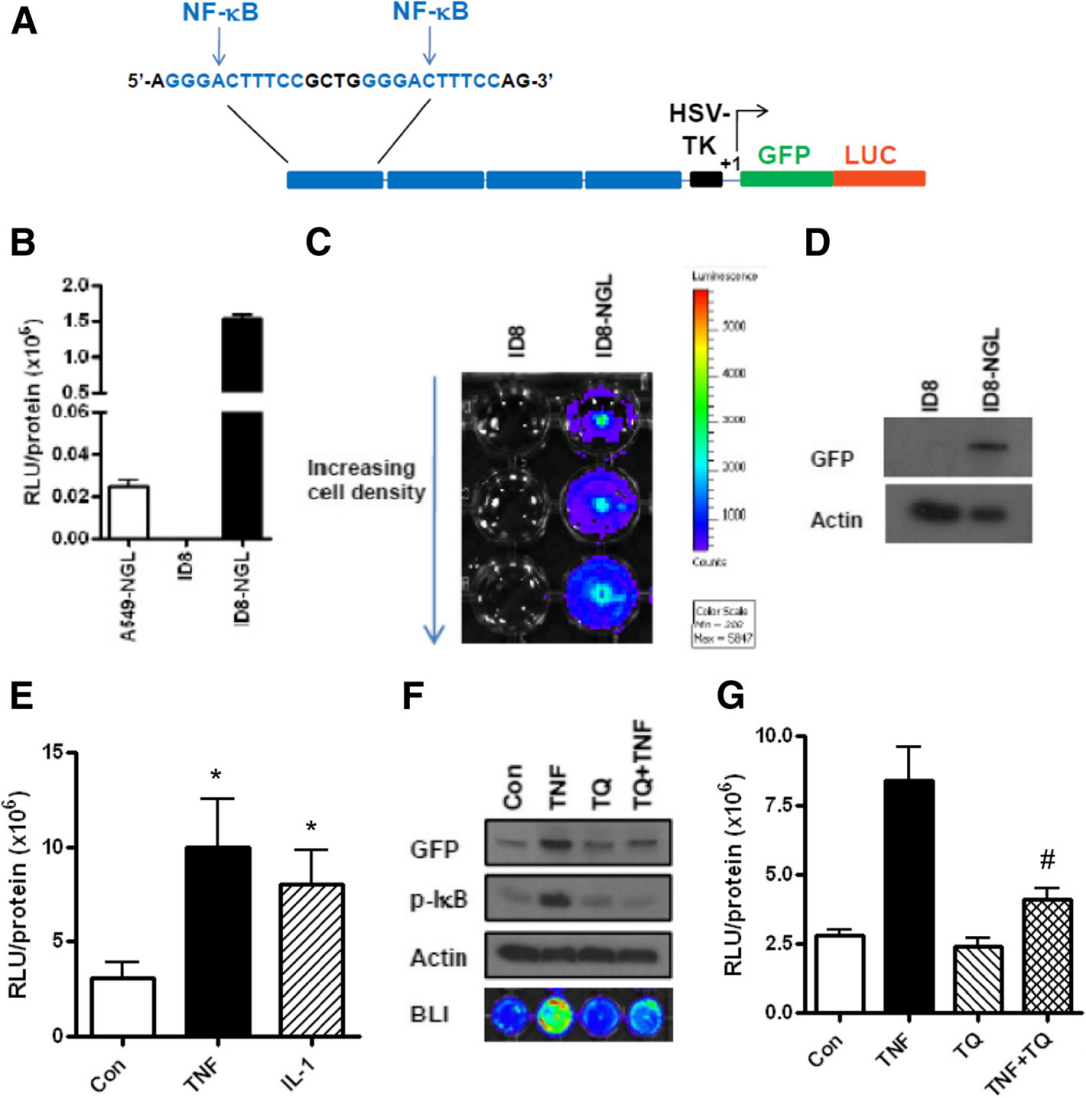
**Characterization of ID8-NGL cells. (A)** Schematic diagram of the pEGFPluc NGL reporter plasmid. The plasmid contains 4 tandem copies of a 36 base pair enhancer of the HIV long terminal repeat, which contains 2 consensus NF-κB binding sites (blue). Basal NF-κB reporter activity in ID8-NGL cells compared to parental ID8 cells was measured in **(B)** luciferase assays from protein extracts and **(C)** by bioluminescence imaging of cell monolayers. **(D)** Western blot showing GFP expression in ID8-NGL cells not observed in parental ID8 cells. Actin was used as the loading control. **(E)** Stimulatory effect of a 4 h treatment with TNF-α and IL-1β (both 10 ng/ml) on luciferase activity in protein extracts from ID8-NGL cells. **(F)** Western blot showing the stimulatory effect of TNF-α on GFP and p-IκB expression after 4 h is inhibited by 2 hours’ pre-treatment with the NF-κB inhibitor, thymoquinone (50 μM). Bioluminescent imaging is also shown. **(G)** Stimulatory effect of TNF-α on luciferase activity was also inhibited by thymoquinone (50 μM). Values are mean + SD of 3 independent experiments. * p < 0.01 relative to Control, # p < 0.01 relative to TNF alone, both Student’s t test.

Mouse epithelial ovarian cancer (ID8) cells are a well-established cell line derived from C57BL/6 mice that are routinely used in syngeneic mouse models of ovarian cancer [4,11,27,28]. Here, we have generated ID8 cells stably expressing an NF-κB-dependent reporter plasmid, termed ID8-NGL. To generate the NGL plasmid, four tandem copies of the 36-base enhancer from the 5′ HIV-long terminal repeat, each containing two NF-κB binding sites GGGACTTTCC, were cloned into the pEGFPluc vector backbone [25,26] (Figure 1A).

The NGL plasmid was transfected into ID8 cells according to manufacturer’s instructions using Lipofectamine 2000 (Invitrogen, Carlsbad, CA). Clones with genomic incorporation of plasmid DNA were selected by growth in 10% fetal bovine serum (FBS)-supplemented DMEM High-Glucose medium (Invitrogen) with 400 μg/ml G418 (Sigma Chemical Co., St Louis, MO). Clones were selected using an inverted light microscope and initially tested for NGL expression in luciferase assays. Further validation of ID8-NGL clones with basal luciferase activity greater than 1×10^6^ RLU/mg protein was performed by bioluminescence imaging of cell cultures and Western Blot detection of GFP expression.

The ID8-NGL clone selected for *in vivo* experiments was cultured in 10% FBS-supplemented DMEM High-Glucose medium with 400 μg/ml G418, and passaged by standard techniques. Parental ID8 cells and lung epithelial cells stably transfected with the NGL plasmid (A549-NGL), a kind gift of Dr. Timothy Blackwell (Vanderbilt University, Nashville, TN) [29], were used for comparison in these characterization studies. Finally, we confirmed that the ID8-NGL clone selected responded appropriately to classical activators of NF-κB signaling, TNF-α and IL-1β (R&D Systems, Minneapolis, MN), and the NF-κB inhibitor, thymoquinone (Sigma).

### Animal model and drug treatment

Wild-type C57BL/6 mice were injected intra-peritoneally (IP) with 1x10^7^ ID8-NGL cells in 200 μl PBS or mock-injected with an equal volume of sterile PBS. 21 gauge safety hypodermic needles (Becton, Dickinson and Company, Franklin Lakes, NJ) were used for these injections. Tumor cells were allowed to grow in mice for up to 90 days before humane sacrifice. A subset of mice underwent bioluminescence imaging at defined time points. At 30 days after ID8-NGL injection, a subset of mice (5 per group) was randomly selected to receive thrice weekly 20 mg/kg thymoquinone or PBS vehicle by IP injection for 10 days [30]. No signs of drug toxicity were observed in the TQ-treated mice.

Tumor progression was also monitored by body weight and abdominal girth measurements. At time of sacrifice, abdominal ascites fluid was extracted with hypodermic syringe, and volume measured. If no measurable ascites was present, peritoneal lavages were performed by injecting 8 ml PBS intra-peritoneally and carefully extracting the fluid with a hypodermic syringe. Tumor implants in the peritoneal wall and the mesentery were harvested and snap frozen or formalin-fixed for further analysis. The experimental protocol was reviewed and approved by the Institutional Animal Care and Use Committee at Vanderbilt University.

### Luciferase assays

Luciferase activity was measured in harvested tumors following tissue homogenization in 1 ml passive lysis buffer, and in whole cell protein extracts from cells grown *in vitro*, using the Promega Luciferase Assay system (Madison, WI). Activity was analyzed using a GloMax Luminometer (Promega). Results were expressed as relative light units (RLU) normalized for protein content, as measured by the Bradford assay (Bio-Rad, Hercules, CA).

### Bioluminescence imaging

Mice were anesthetized and luciferin (Biosynth AG, Rietlistr, Switzerland) administered by retro-orbital injection (1 mg/mouse in 100 μl isotonic saline). Mice were imaged using a Xenogen IVIS 2000 imaging device (Caliper Life Sciences., Hopkinton, MA). Light emission was detected by an ICCD camera and quantified using image processing Living Image v 2.5 software (Caliper Life Sciences). For bioluminescence of cell cultures, a final concentration of 150 μg/ml luciferin was added to cells just prior to imaging with the Xenogen IVIS 2000.

### Analysis of ascites/peritoneal lavage fluid

Ascites or peritoneal lavage fluid was centrifuged at 1500 rpm for 5 minutes to separate cells from supernatant. Where applicable, red blood cells were lysed by ACK lysing buffer according to manufacturer’s instructions (Invitrogen, Carlsbad, CA). An aliquot of cells were suspended in PBS with 1% BSA for total cell counts using a grid haemocytometer. Cells were then either snap-frozen for RNA extraction, or centrifuged onto microscope slides using a Thermo Cytospin II Cytocentrifuge (500 rpm for 10 minutes) for differential counts of inflammatory cells in hematoxylin and eosin-stained cells or immuofluorescence analysis.

### RNA extraction and quantitative RT-PCR

RNA from ascites fluid or peritoneal lavages was isolated using the RNeasy Mini kit (Qiagen, Valencia, CA). DNase-treated samples were subjected to real-time PCR using SYBR Green PCR Master Mix (Applied Biosystems, Foster City, CA). Steady-state mRNA levels of the M1 macrophage mark, CC chemokine ligand 3 (CCL3), and M2 macrophage mark, mannose receptor (mann-R) were expressed relative to corresponding GAPDH levels using the comparative 2^ΔΔCt^ method [31]. Relative expression values were also normalized to levels of the epithelial marker cytokeratin-18 (CK18) to account for the epithelial (tumor) cell component of ascites or peritoneal lavage fluid. Primers sequences used were:

Cytokeratin-18 (CK18): forward: 5′- ACGGAGCTGAGACGCACCCT-3′, reverse: 5′–GCCTCCACATCCCCGAGGCT-3′;

CC chemokine ligand 3 (CCL3): forward: 5′-TGCCCTTGCTGTTCTTCTCT-3′, reverse: 5′–GATGAATTGGCGTGGAATCT-3′;

Mannose receptor (mann-R): forward: 5′-CAAGGAAGGTTGGCATTTGT-3′, reverse: 5′-CCTTTCAGTCCTTTGCAAGC-3′;

GAPDH: forward: 5′-TGAGGACCAGGTTGTCTCCT-3′, reverse: 5′-CCCTGTTGCTGTAGCCGTAT-3′.

### Immunofluorescence/immunohistochemistry

Processing, embedding and sectioning of formalin-fixed ID8-NGL tumor tissue, and hematoxylin and eosin staining for histology, were performed in The Allergy/Pulmonary & Critical Care Med Division Immunohistochemistry Core at Vanderbilt [32]. Immunofluorescent analysis of formalin-fixed paraffin-embedded tumor tissue was performed using standard deparaffinization and rehydration in 100%-90%-80%-70% ethanol. To reduce tissue autofluorescence, the samples were incubated in 0.5% Sudan Black in 70% ethanol for 10 min, 70% ethanol without Sudan Black for 10 min, and 50% ethanol for 2 min. The samples were permeabilized in 0.4% Triton X-100 for 10 min and subjected to antigen retrieval by boiling in 10 mM sodium citrate for 7 min. The following primary antibodies were used: rabbit polyclonal anti-Ki67/Mib-1 (Abcam, Cambridge, UK; 1:200 dilution), goat polyclonal anti-GFP (Abcam, 1:200), mouse monoclonal anti-pan-cytokeratin (Abcam, 1:100), and rabbit polyclonal anti-phospho-p65 (serine 276) (Santa Cruz Biotechnology, Dallas, TX, 1:100. Secondary antibodies used were goat anti-rat or -rabbit or -mouse conjugated with Molecular Probes Alexa488 or Alexa594 (Invitrogen). After staining with secondary antibodies, the slides were washed 3 times with TBS-Tween20, then washed once in TBS and then stained with Molecular Probes TO-PRO-3 (Invitrogen) for 15 min (1 μM in TBS). The slides were washed with TBS and the coverslips mounted using Molecular Probes ProlongGold antifade reagent (Invitrogen). Images were acquired using an LSM 510 Meta confocal microscope in the Vanderbilt University Medical Center Imaging Core.

For ascites fluid, macrophages and tumor cells were identified by immunofluorescent staining for F4/80 (AbD Serotec; Raleigh, North Carolina, 1:200) and pan-cytokeratin, respectively (Abcam, 1:1000) as described [32]. Secondary antibodies used were as described above. The percentage of F4/80-positive/CK-negative cells was counted in 3 separate fields at 40× using an immunofluorescent microscope. At least 200 cells were counted per sample.

Immunohistochemical analysis of PAX8 and F4/80 expression in tumor tissue was performed as described [32], using rabbit polyclonal anti-PAX8 (Proteintech, Chicago, IL,1:50) and rat polyclonal anti-F4/80 (AbD Serotec; 1:50), respectively.

### Western blotting

Whole cell protein isolation, subcellular fractionation, western blotting and signal detection and quantification were performed as described previously [33,34]. Primary antibodies used were mouse monoclonal anti-GFP (Invitrogen), mouse monoclonal anti-phospho-IκB (Cell Signaling Technology, Danvers, MA; 1:500), rabbit polyclonal anti-p65 (Cell Signaling Technology; 1:1000), mouse monoclonal anti-histone H3 (Millipore; 1:1000) and anti-β-actin (Sigma; 1:10000).

### Statistical analysis

Unless otherwise indicated, values shown for *in vitro* experiments were the mean + SD of 3 independent experiments, with comparison of groups performed by 2-tailed Student’s t test. Comparison of groups in *in vivo* experiments was performed by 2-tailed Mann-Whitney test. A p value < 0.05 is considered statistically significant. The relationship between BLI and indices of tumor burden was analyzed by Spearman correlation.

## Results

### Effects of stimulation or inhibition of NF-κB can be quantified in ID8 mouse ovarian cancer cells stably expressing the NF-κB reporter (ID8-NGL)

Mouse epithelial ovarian cancer (ID8) cells are a well-established cell line derived from C57BL/6 mice that are routinely used in syngeneic mouse models of ovarian cancer [4,11,27,28]. Here, we generated ID8 cells stably expressing an NF-κB-dependent reporter plasmid, termed ID8-NGL. The NGL plasmid expresses a green fluorescent protein (GFP)/luciferase fusion product under the control of a synthetic NF-κB dependent promoter with a total of 8 NF-κB binding sites [25,26] (Figure 1A). As expected in this tumor cell line, we confirmed detectable baseline levels of luciferase activity (Figure 1B&C) and GFP expression (Figure 1D) in ID8-NGL cells compared to parental ID8 cells. Lung tumor cells (A549-NGL) carrying the same reporter construct were used as a positive control.

To test the ability to measure NF-κB responses, ID8-NGL cells were treated with known activators of NF-κB, TNF-α and IL-1β. Both activators stimulated a significant increase in luciferase activity in protein extracts compared to controls after 4 hours’ treatment (p < 0.01, Student’s t test) (Figure 1E). Results were supported by bioluminescence imaging of cell monolayers (Figure 1F). We then demonstrated the specificity of this effect by the abrogation of TNF-α-mediated stimulation of luciferase activity and GFP expression by 2 hours pre-treatment with the NF-κB inhibitor, thymoquinone (TQ) (Figure 1F&G). In addition, western blot analysis was consistent with the known ability of TQ to inhibit TNF-α induction of IκB phosphorylation (Figure 1F).

### Intra-peritoneal injection of ID8-NGL cells produces ascites and peritoneal carcinomatosis in mice

The parental ID8 cell line for the ID8-NGL cells has been shown to model the features of advanced serous ovarian cancer with the development of malignant, bloody ascites and peritoneal carcinomatosis [27,28,35]. Here, we confirmed that ID8-NGL cells form a similar pattern of ascites and peritoneal spread in our syngeneic model. By 90 days after tumor injection, mice developed prominent abdominal distension indicative of ascites (Additional file 1: Figure S1A). This was consistent with markedly increased body weight and abdominal girth observed in mice at this time point compared to PBS-injected controls (Additional file 1: Figure S1B&C). Careful dissection of the peritoneal cavities revealed that tumor cell-injected mice displayed a reproducible pattern of tumor dissemination. As shown in Additional file 1: Figure S1D tumor nodules were detected embedded in the peritoneal wall (yellow arrows), and in the mesentery of the intestines (white arrows). A representative tumor implant in the smooth muscle of the peritoneal wall is shown in Additional file 1: Figure S1E.

### Increased NF-κB activity is detected during progressive ovarian cancer dissemination in the peritoneal cavity

**Figure 2:**
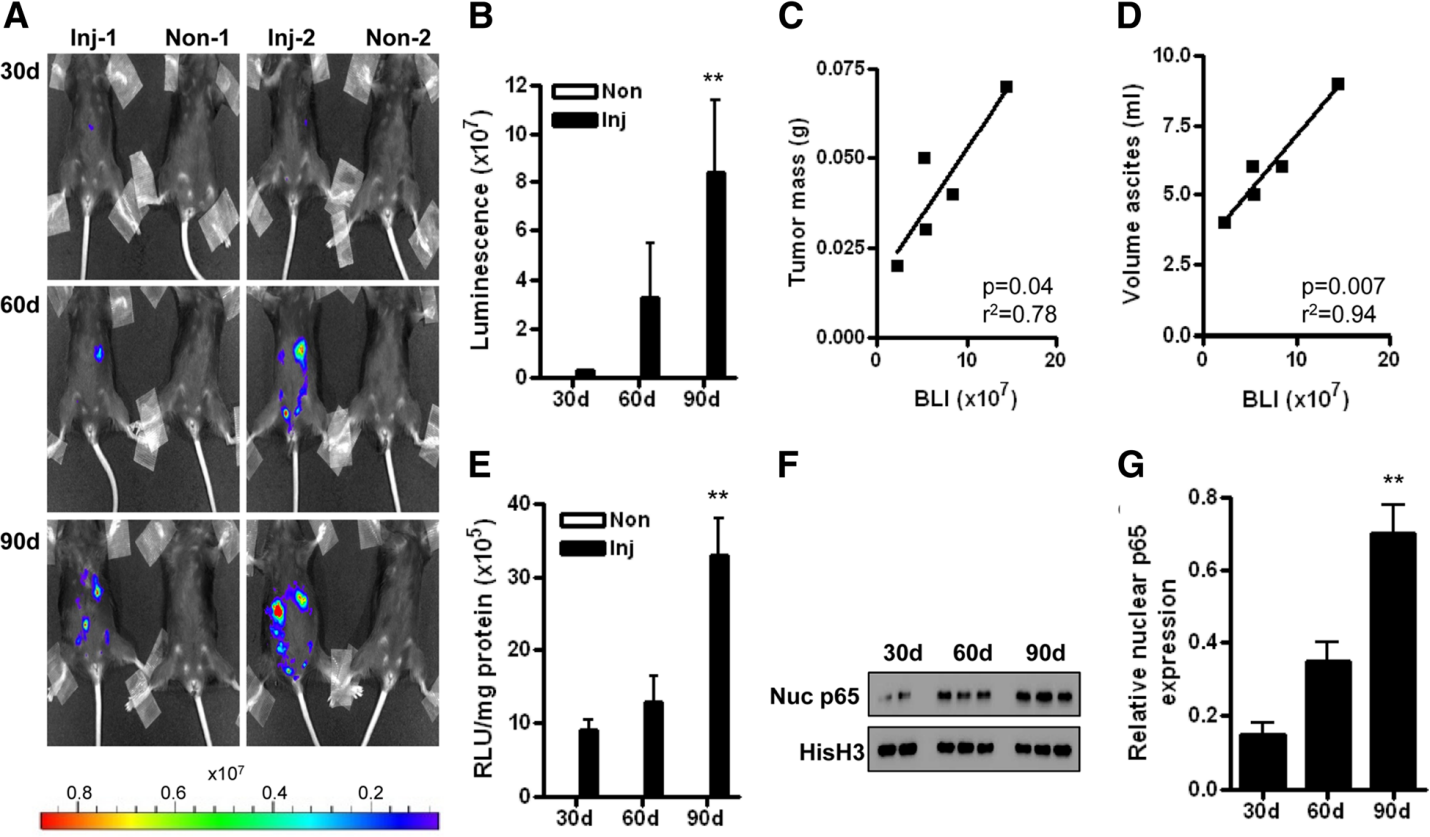
**NF-κB reporter activity increases during ovarian cancer progression. (A)** Representative BLI of WT mice injected with ID8-NGL cells or PBS (non-inject) over 90d following injection showing NF-κB reporter signal in tumor cells. Quantification of BLI in the abdominal cavity is shown in **(B)**. BLI was significantly correlated with **(C)** mesenteric tumor mass and **(D)** volume of ascites at 90d (Spearman). **(E)** Luciferase activity of the NF-κB reporter was measured in harvested tumors and expressed relative to cellular protein. **(F)** Western blot analysis of p65 in nuclear extracts from harvested tumors. Equal loading was shown by probing for the nuclear-specific protein, histone H3. **(G)** Nuclear p65 expression relative to corresponding histone H3 levels was measured by densitometry. Values shown are mean + SD from 5 mice per group. ** p < 0.01 relative to 30d or 60d, Mann-Whitney test.

First, we confirmed that mice injected with ID8-NGL cells displayed high levels of luminescence compared to PBS-injected mice by bioluminescence imaging. Representative images are shown in Figure 2A. When region of interest analysis was performed to quantify abdominal luminescence, ID8-NGL-injected mice displayed approximately 100-fold higher levels than non-injected controls (average of 3.7 ± 2.3 × 10^7^ RLU over the 30, 60 and 90 day time points examined compared to 0.013 ± 0.009 × 10^7^ RLU, respectively, p < 0.001, Mann-Whitney test), indicating the specificity of the signal. NF-κB reporter activity increased in a time-dependent manner (Figure 2B), which showed correlation with indices of tumor burden such as mesenteric tumor mass and ascites volume at 90 days (Figure 2C&D). To differentiate between specific increases in NF-κB activity in tumor cells and an overall increase in NF-κB signal due to increased tumor burden, we performed luciferase assays of snap-frozen tumor tissue. As shown in Figure 2E, there was a specific increase in NF-κB activity within tumor tissue (when corrected for cellular protein) in later phases of peritoneal tumor spread. We confirmed this observation independently of the NF-κB reporter by measuring levels of p65 in nuclear extracts from harvested mesenteric tumors. As shown in Figure 2F&G, the highest levels of nuclear p65 expression were observed in 90 day tumors.

### Peritoneal implants formed by ID8-NGL cells have characteristics representative of high-grade serous epithelial ovarian cancer

**Figure 3:**
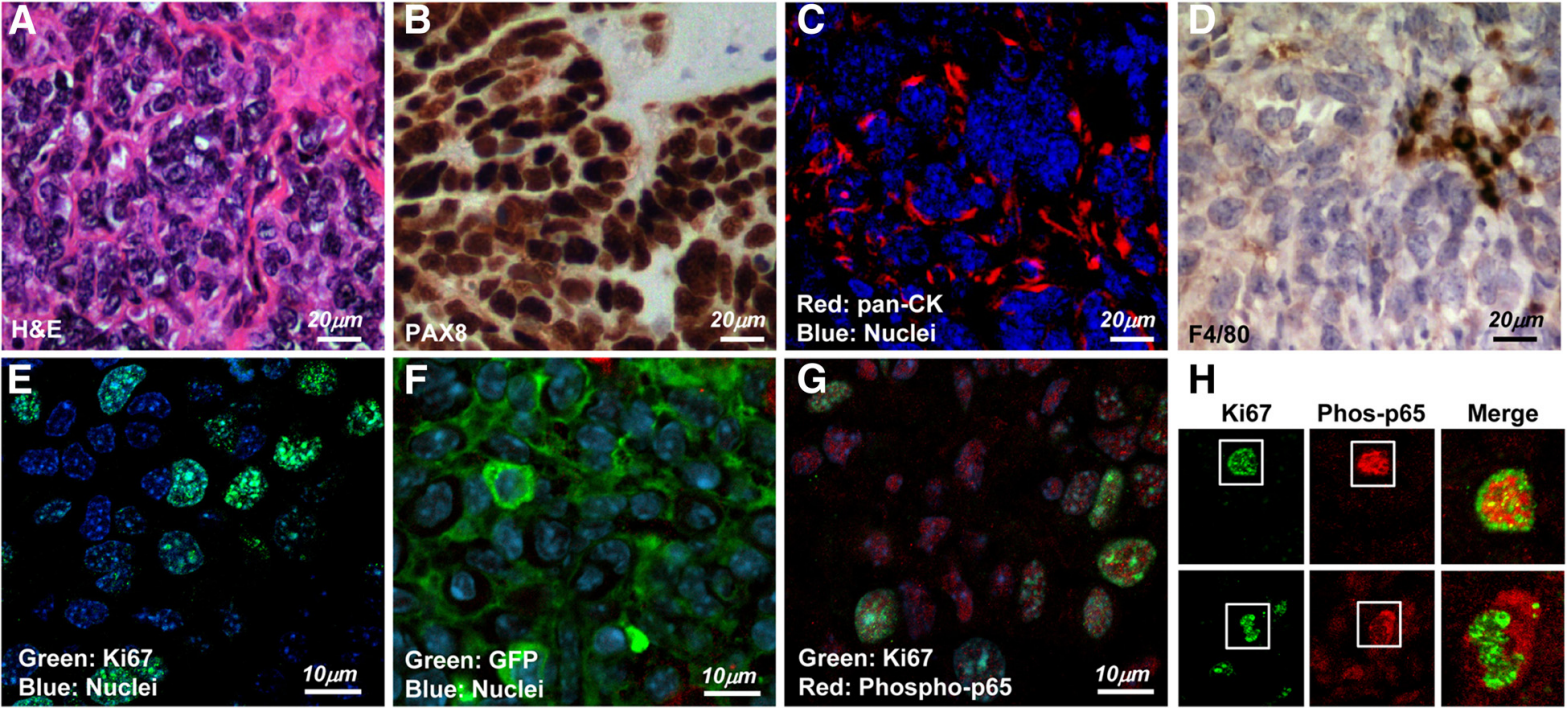
**Molecular characterization of ID8-NGL tumors. (A)** H&E stain of a representative area of ID8-NGL tumors. **(B)** Immunohistochemical detection of the marker of high-grade serous ovarian tumors, PAX8. **(C)** Fluorescent microscopy showing expression of the epithelial marker pan-cytokeratin (CK) expression relative to TO-PRO-3-stained nuclei. **(D)** Immunohistochemical detection of the mature macrophage marker, F4/80, in tumor sections. High power (×100) confocal images of the representative tumor section showing immunofluorescent detection of **(E)** the proliferation marker Ki67/mib-1, **(F)** the GFP reporter and **(G)** overlap of Ki67/mib-1 and phospho-p65 (serine 276) (red) in the epithelial component of the tumor. TO-PRO-3-stained nuclei are in blue. **(H)** Co-expression of phospho-p65 (red) and Ki67/mib-1 (green) is observed in the nuclei of a subset of cells. The panels on the right show merged images of the corresponding boxed areas for two representative nuclei.

To determine if ID8-NGL tumors were representative of high-grade serous epithelial ovarian cancer, we first performed H&E staining and confirmed that tumors displayed the characteristic morphology of high-grade serous cancer (Figure 3A). Furthermore, we stained tumors for expression of PAX8, an established marker of high-grade serous ovarian tumors [36]. We found strong nuclear immunoreactivity for PAX8 in epithelial tumor cells, but not in the stroma, as confirmed by staining for the epithelial marker, cytoplasmic pan-cytokeratin (Figure 3B&C). Macrophage infiltration in stromal areas of the tumor was also demonstrated by staining with F4/80, a marker of mature macrophages (Figure 3D).

We then performed immunofluorescent analyses of molecular markers of proliferation, NGL reporter expression, and NF-κB signaling in the epithelial component of tumors. Staining for Ki67/mib-1 demonstrated that the tumors were highly proliferative (Figure 3E). Activity of the NGL reporter in tumors was shown by detection of the GFP component of the fusion reporter protein (Figure 3F), and NF-κB activity in tumors was independently confirmed by analysis of nuclear expression of phosphorylated-p65 (serine 276) (Figure 3G). We then demonstrated that phospho-p65 and Ki67 were co-expressed in the nuclei of a subset of tumor cells, but that there was minimal overlap between the two within the nuclei (Figure 3H). This is consistent with the localization of Ki67 to ribosomal RNA-encoding nucleoli in proliferating cells [37].

### Populations of monocytes and mature macrophages increase in association with tumor load within the peritoneal cavity

**Figure 4:**
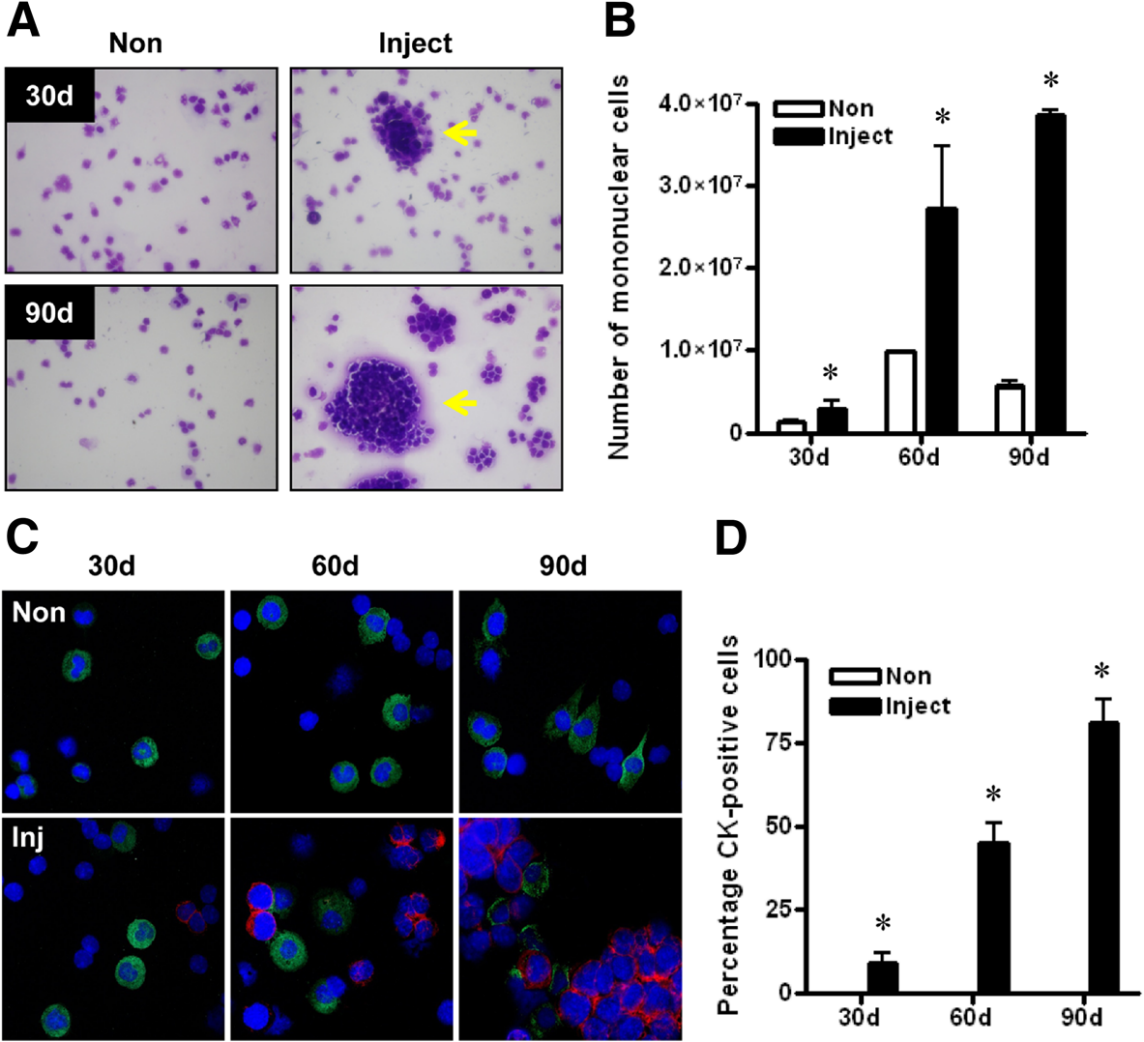
**Analysis of fluid collected from the peritoneal cavity. (A)** Representative images of cytospin analyses of peritoneal lavage fluid/ascites collected from mice injected with tumor cells (Inject) or mock-PBS-injected mice (Non). Tumor cell clumps are shown (yellow arrow). **(B)** Overall number of mononuclear cells collected increased in mice with tumors and with duration of progression. **(C)** Expression of the epithelial marker, pan-cytokeratin (CK; red), and the mature macrophage marker, F4/80 (green), in ascites fluid on cytospin slides. Percentage of CK-positive cells is shown in **(D)**. Values are mean + SD of counts from 200 cells from each of 3 mice per group. * p < 0.01 relative to non-inject, Mann-Whitney test.

To assess the composition of peritoneal cell populations prior to the development of ascites and during early phases of peritoneal spread, we performed peritoneal lavages with sterile PBS to collect cells from injected mice or control non-injected mice. Cytospin counts of H&E-stained slides revealed large cell clusters without definable borders consistent with tumor cells in the peritoneal fluid (see arrows in Figure 4A), which were excluded from our inflammatory cell counts. Mononuclear cells were the predominant inflammatory cell population present in the peritoneal cavities of both non-injected and injected mice. Furthermore, in injected mice mononuclear cells made up consistently 90% or greater of the inflammatory cells harvested in ascites fluid or in peritoneal lavages irrespective of duration of exposure to tumor cells (data not shown). The overall number of mononuclear cells harvested was elevated approximately 8-fold relative to non-injected controls at 90 days in the presence of tumor cells and with increasing duration of tumor spread (approximately 15-fold increase from the 30 day to 90 day time point in injected mice) (Figure 4B). Since macrophages are a major subset of mononuclear cells, and given the established role of peritoneal macrophages in ovarian cancer progression [4], we also performed immunofluorescent analysis of ascites fluid to detect epithelial (tumor) cells and mature macrophages with antibodies against pan-cytokeratin and F4/80, respectively (Figure 4C). These analyses confirmed that the tumor cells were a major component of ascites fluid at later time points (Figure 4D), and that mature macrophages account for at least 60% of the non-epithelial cells present. Collectively, these results suggest an elevated inflammatory response is associated with the presence of tumor cells in the peritoneal cavity.

**Figure 5:**
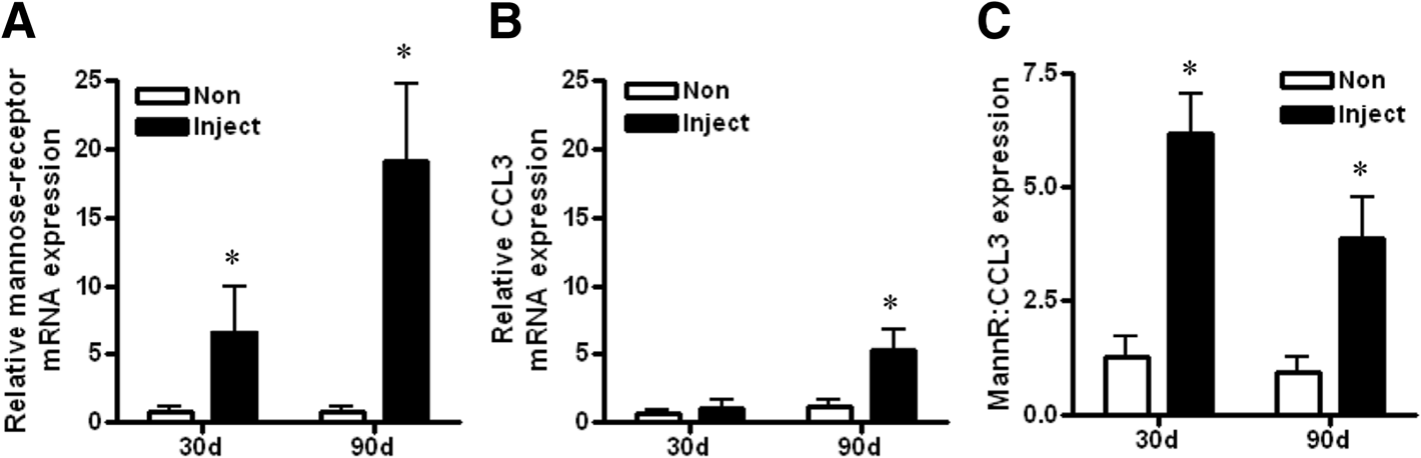
**Elevation of M2 macrophage marker in tumor-bearing mice.** QPCR analysis of the mRNA expression of the markers of **(A)** M2 macrophages, mannose-receptor (mann-R) and **(B)** M1 macrophages, CCL3, in RNA extracted from peritoneal lavages or ascites fluid. Values were normalized to corresponding levels of GAPDH mRNA expression and also CK18 mRNA levels, to account for epithelial content. The expression ratio of mann-R to CCL3 is shown in **(C)**. Values are mean + SD for 3 mice per group. * p < 0.01 relative to non-injected mice, Mann-Whitney test.

We also examined expression of markers of cytotoxic, anti-tumor M1 and pro-tumor M2 macrophages in RNA extracted from peritoneal lavage fluid or ascites. CC chemokine ligand 3 (CCL3) and mannose-receptor (mann-R) are well-established markers of M1 and M2 macrophages, respectively [38]. Expression of mann-R, was significantly increased in injected mice compared to non-injected controls at both the 30 day and 90 day time points, with an approximately 20-fold increase at 90 days (Figure 5A). There was a markedly smaller increase in CCL3 expression at 90 days in tumor-bearing mice (approximately 5-fold), with no significant difference observed at 30 days (Figure 5B). Consistent with these observations, the ratio of mann-R to CCL3 expression was significantly higher in injected mice at both time points (Figure 5C).

### Reduced NF-κB activity in tumors is associated with reduced expression levels of mannose-receptor in ascites fluid

**Figure 6:**
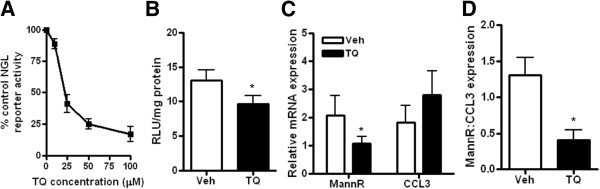
**Thymoquinone reduces NF-κB activity in tumors and expression of M2 macrophage marker in ascites fluid. (A)** Effects of increasing concentrations of TQ on NF-κB reporter activity in ID8-NGL cells after 24 hours’ treatment as measured in luciferase assays. **(B)** Effect of 10 days treatment with 20 mg/kg TQ or PBS vehicle (thrice weekly IP injections) on NF-κB reporter activity in harvested ID8-NGL tumors. **(C)** QPCR analysis of the mRNA expression of the markers of M2 macrophages, mannose-receptor (mann-R) and M1 macrophages, CCL3, in RNA extracted from peritoneal lavages. Values were normalized to corresponding levels of GAPDH mRNA expression and also CK18 mRNA levels, to account for epithelial content. The expression ratio of mann-R to CCL3 is shown in **(D)**. Values are mean + SD for 5 mice per group. * p < 0.01 relative to vehicle, Mann-Whitney test.

To determine whether modulation of NF-κB activity in tumors could alter the macrophage characteristics in ascites fluid, we treated ID8-NGL-injected mice with the NF-κB inhibitor, thymoquine (TQ). Initial validation experiments confirmed that there was a concentration-dependent reduction in NGL reporter activity in ID8-NGL treated with TQ *in vitro* (Figure 6A). 30 days after ID8-NGL injection, mice were injected IP with TQ or PBS vehicle thrice weekly for 10 days. Because of the limited amount of macroscopic tumor observed at the time of sacrifice, we were unable to accurately quantify drug effects on tumor burden. Furthermore, as expected, no ascites was observed at this relatively early stage of tumor progression. We prioritized snap-freezing tumors for luciferase assays, and determined that TQ treatment reduced NGL reporter activity by approximately 25% in tumors in luciferase assays compared to vehicle-treated tumors (Figure 6B).

Reduced NF-κB activity with TQ treatment was associated with significantly reduced levels of the M2 macrophage marker mann-R (48.3 ± 5.1% compared to vehicle, p < 0.02, Mann-Whitney test) (Figure 6C) in peritoneal lavage fluid, reflected in a significant reduction in the mannR:CCL3 expression ratio (Figure 6D). Therefore, this study provides evidence that macrophage populations may be shifted towards an “anti-tumor” M1 phenotype by reducing NF-κB activity in tumor cells.

## Discussion

In this study, we have modified the ID8 syngeneic mouse model of ovarian cancer to allow intra-vital tracking of NF-κB activity, known to play a critical role in development of ascites and peritoneal dissemination [4,13-16], during tumor progression. The ID8-NGL reporter cells are an important tool for building upon our knowledge of the role of NF-κB in ovarian cancer spread, including the link between NF-κB activity in tumors and recruitment of host immune macrophages within the peritoneal cavity. The relationship between tumor progression and the immune system cannot be fully addressed by clinically useful xenograft models of human ovarian cancer, because those models require the use of immunocompromised mice. Therefore, the ID8-NGL immunocompetent model has potential clinical relevance because the development of ascites, peritoneal carcinomatosis and macrophage infiltrates recapitulate advanced human ovarian cancer [27,28,35].

Here, we show that ID8-NGL reporter cells show the appropriate response to NF-κB activation and inhibition *in vitro*, and to the NF-κB inhibitor, TQ, *in vivo*. We have also demonstrated that there was markedly increased NF-κB activation in ID8-NGL cells during the later stage of tumor progression, which was associated with rapid ascites accumulation and tumor dissemination. Furthermore, NF-κB activation was directly associated with cell proliferation in a subset of tumor cells *in vivo*. These results are consistent with previously published results [12,39,40]. We were unable to independently confirm our *in vivo* bioluminescence results by fluorescent imaging of ID8-NGL tumors, due to insufficiently strong GFP fluorescence of the fusion reporter protein. However, GFP expression was detectable by alternative techniques, such as western blot and indirect immunofluorescence, and these results were consistent with our bioluminescence and luciferase assay data.

While other groups have focused on immune cell infiltration into tumors [22-24], we studied the immune cell population in ascites and peritoneal lavage fluid. We found that macrophages dominate the immune cell population in the peritoneal cavity, and the number of macrophages increases in parallel with the progression of ascites and peritoneal tumor dissemination. Furthermore, M2 and to a lesser extent M1 markers of tumor-associated and cytotoxic macrophage phenotypes, respectively, increase with peritoneal tumor spread in ovarian cancer. An area of ongoing investigation is to determine how activity of NF-κB in tumor cells influences macrophages and macrophage phenotypes in promoting ovarian cancer spread. We believe that this ID8-NGL model serves as an important system for addressing this question. As proof-of-principle, we demonstrated that chemical reduction of NF-κB activity in tumors was associated with reduced levels of the M2 macrophage mark, mannose-receptor, in ascites fluid. Although specific inflammatory cell populations were not dissected, these results provide evidence that macrophage populations may be shifted towards an “anti-tumor” M1 phenotype by reducing tumor NF-κB activity with a systemic NF-κB inhibitor. Furthermore, the ID8-NGL model could serve as a powerful tool for pre-clinical testing of agents that target NF-κB, such as TQ, in future intervention studies.

We acknowledge that a limitation of our NF-κB-linked reporter model is that bioluminescent imaging does not provide a direct measure of the total number of tumor cells, in contrast to imaging of tumors derived from ID8-Luc cells, which have constitutive stable expression of the luciferase reporter [4]. However, luciferase assays on harvested tumors where cellular content can be normalized confirmed strongly increased NF-κB reporter activity at late-stage progression. This suggests that additional insight can be gained by comparison of live imaging with data from isolated tumor cell populations. Other non-invasive imaging modalities, performed in parallel to bioluminescence imaging, such as microPET/CT or intra-vital imaging of fluorescent dyes [35], are alternative options to match NF-κB activity with tumor burden in the ID8-NGL model.

## Conclusions

We have generated mouse ID8 ovarian cancer cells stably expressing the NGL NF-κB reporter plasmid to track NF-κB activity in a syngeneic mouse model. The strengths of this model are (i) the capacity to evaluate the role of NF-κB activity in mediating the link between cancer progression and the immune system in ovarian cancer, and (ii) the ability to measure NF-κB activity through a dual luciferase -GFP fusion protein, allowing multiple independent assays to confirm changes in NF-κB activity in living animals and in harvested tumors. Additional applications for this model include pre-clinical development of drugs that target NF-κB, such as NF-κB inhibitors, in ovarian cancer.

## Abbreviations

NF-κB: Nuclear factor-kappaB; NGL: NF-κB-GFP-Luciferase; WT: Wild type.

## Competing interests

The authors disclose no competing interests.

## Authors’ contributions

AJW characterized ID8 cells stably expressing the NGL reporter, oversaw the experiments, performed dissections and collection of ascites/peritoneal lavages, performed tumor cell injections, analyzed the data, and drafted the manuscript. LC was responsible for husbandry C57BL/6 mice, and performed bioluminescence imaging and cytospin counts. WB was responsible for mouse husbandry, and performed bioluminescence imaging and cytospin counts. JS maintained cell lines, performed bioluminescence imaging, and processed ascites/peritoneal lavage fluid for RNA and protein extraction. OT performed IHC and IF on tissue samples. HJL helped generate ID8-NGL cells. FY conceived the study, provided the C57BL/6 mice, and consulted on experimental design. DK conceived the study, consulted on experimental design and data analysis, and drafted the manuscript. All of the authors have read and approved the final version.

## Supplementary Material

Additional file 1: Figure S1 Peritoneal dissemination of ID8-NGL cells during tumor progression. (A) Mice injected with ID8-NGL cells show distended abdomen indicative of ascites formation at 90d after injection compared to PBS-injected mice (non-inject). Changes in (B) body weight and (C) abdominal girth were measured. Values are mean + SD for 5 mice per group. (D) Mice injected with ID8-NGL cells display abdominal dissemination of tumor cells. The main sites of tumor implantation are the peritoneal wall (yellow arrows) and in the mesentery (white arrow). (E) H&E staining example of tumor implantation in the peritoneal wall. Click here for file
