# Supplementary material for: Tracking NF-κB activity in tumor cells during ovarian cancer progression in a syngeneic mouse model
Source: J Ovarian Res. 2013 Sep 10;6:63. doi: 10.1186/1757-2215-6-63 (PMC3846584; doi:10.1186/1757-2215-6-63)
Supplement: Additional file 1: Figure S1 — Peritoneal dissemination of ID8-NGL cells during tumor progression. (A) Mice injected with ID8-NGL cells show distended abdomen indicative of ascites formation at 90d after injection compared to PBS-injected mice (non-inject). Changes in (B) body weight and (C) abdominal girth were measured. Values are mean + SD for 5 mice per group. (D) Mice injected with ID8-NGL cells display abdominal dissemination of tumor cells. The main sites of tumor implantation are the peritoneal wall (yellow arrows) and in the mesentery (white arrow). (E) H&E staining example of tumor implantation in the peritoneal wall. [file 1757-2215-6-63-S1.pdf]

Wilson et al: Supplementary Figure 1

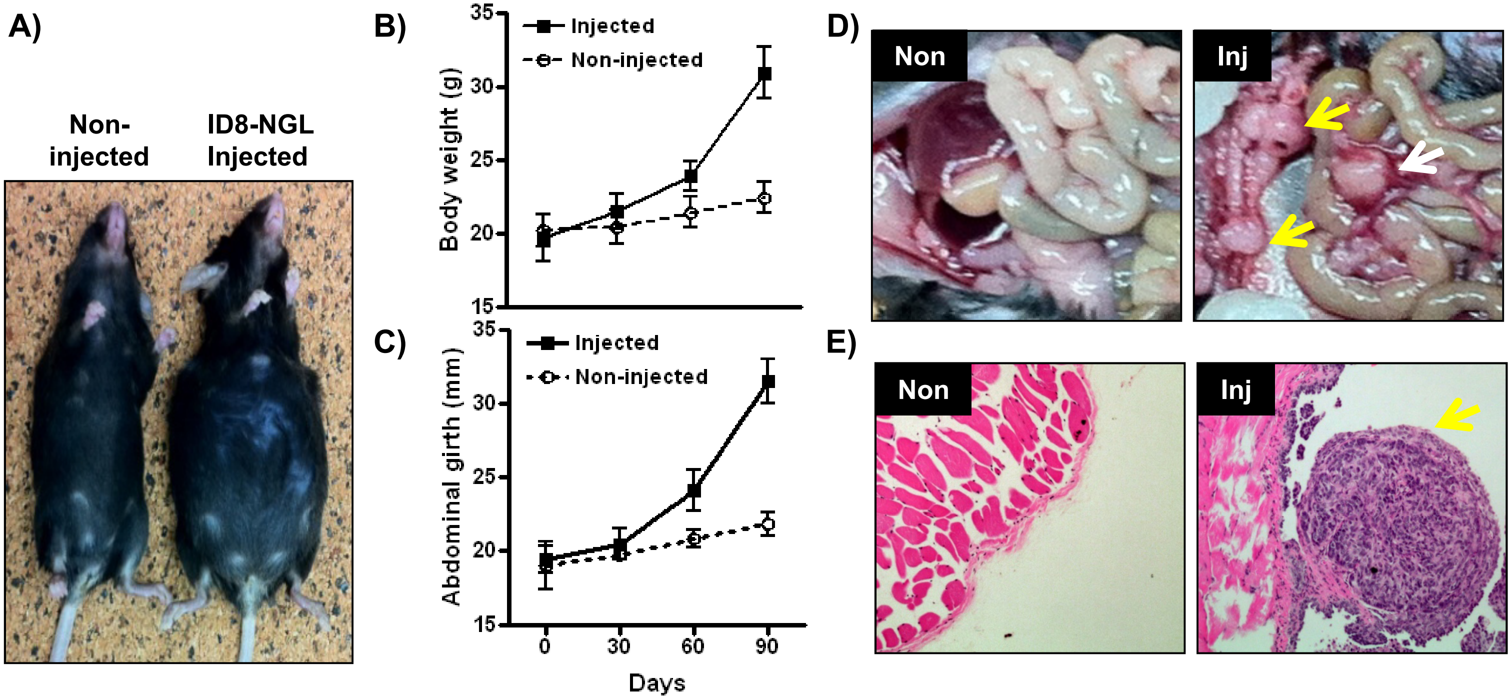

Peritoneal dissemination of ID8-NGL cells during tumor progression. (A) Mice injected with ID8-NGL cells show distended abdomen indicative of ascites formation at 90d after injection compared to PBS-injected mice (non-inject). Changes in **(B)** body weight and **(C)** abdominal girth were measured. Values are mean+SD for 5 mice per group. **(D)** Mice injected with ID8-NGL cells display abdominal dissemination of tumor cells. The main sites of tumor implantation are the peritoneal wall (yellow arrows) and in the mesentery (white arrow). **(E)** H&E staining example of tumor implantation in the peritoneal wall.
